# Review: The Nose as a Route for Therapy. Part 2 Immunotherapy

**DOI:** 10.3389/falgy.2021.668781

**Published:** 2021-07-01

**Authors:** Yorissa Padayachee, Sabine Flicker, Sophia Linton, John Cafferkey, Onn Min Kon, Sebastian L. Johnston, Anne K. Ellis, Martin Desrosiers, Paul Turner, Rudolf Valenta, Glenis Kathleen Scadding

**Affiliations:** ^1^Department of Respiratory Medicine, Faculty of Medicine, Imperial College Healthcare NHS Trust, Imperial College London, London, United Kingdom; ^2^Center for Pathophysiology, Infectiology and Immunology, Institute of Pathophysiology and Allergy Research, Medical University of Vienna, Vienna, Austria; ^3^Division of Allergy and Immunology, Department of Medicine, Queen's University, Kingston, ON, Canada; ^4^Allergy Research Unit, Kingston Health Sciences Centre (KHSC), Kingston, ON, Canada; ^5^Faculty of Medicine, National Heart and Lung Institute, Imperial College London, London, United Kingdom; ^6^Department of Otorhinolaryngologie, The University of Montreal Hospital Research Centre (CRCHUM), Montreal, QC, Canada; ^7^Division of Immunopathology, Medical University of Vienna, Vienna, Austria; ^8^Royal National Ear Nose and Throat Hospital, University College London Hospitals NHS Foundation Trust, London, United Kingdom; ^9^Division of Infection and Immunity, Faculty of Medical Sciences, University College London, London, United Kingdom

**Keywords:** interferon, rhinovirus, microbiome, vaccination, allergen immunotherapy, epithelial barrier, ICAM-1, allergen-specific antibody

## Abstract

The nose provides a route of access to the body for inhalants and fluids. Unsurprisingly it has a strong immune defense system, with involvement of innate (e.g., epithelial barrier, muco- ciliary clearance, nasal secretions with interferons, lysozyme, nitric oxide) and acquired (e.g., secreted immunoglobulins, lymphocytes) arms. The lattice network of dendritic cells surrounding the nostrils allows rapid uptake and sampling of molecules able to negotiate the epithelial barrier. Despite this many respiratory infections, including SARS-CoV2, are initiated through nasal mucosal contact, and the nasal mucosa is a significant “reservoir” for microbes including *Streptococcus pneumoniae, Neisseria meningitidis and SARS -CoV-2*. This review includes consideration of the augmentation of immune defense by the nasal application of interferons, then the reduction of unnecessary inflammation and infection by alteration of the nasal microbiome. The nasal mucosa and associated lymphoid tissue (nasopharynx-associated lymphoid tissue, NALT) provides an important site for vaccine delivery, with cold-adapted live influenza strains (LAIV), which replicate intranasally, resulting in an immune response without significant clinical symptoms, being the most successful thus far. Finally, the clever intranasal application of antibodies bispecific for allergens and Intercellular Adhesion Molecule 1 (ICAM-1) as a topical treatment for allergic and RV-induced rhinitis is explained.

## Intranasal Interferons in Prevention and Treatment of Viral Respiratory Illnesses

Interferons (IFNs) are a family of cytokine mediators with unique immune-modulatory antiviral and anti-proliferative properties which has led to their investigation as treatment and prevention against common colds. Early studies using prophylactic systemic high-dose IFN-α have mostly demonstrated success against rhinoviral colds but had varying efficacy as prophylaxis for other respiratory viruses. Subsequently, use of IFN-α for common colds was halted due to adverse effects. Nasal IFNs may provide similar efficacy with reduced side effects. Some studies using intranasal IFN-γ have demonstrated inefficacy as prophylaxis against colds. Intranasal IFN-λ has not been studied in man against common cold viruses but has shown promising *in-vitro* and mouse-model results. Recent studies investigating IFNs as treatment for virally- induced asthma exacerbations demonstrated improvement in some clinical outcomes. Currently IFNs are being investigated for their use in asthma, COPD and the SARS-CoV-2 pandemic.

Interferons (IFNs) consist of a family of cytokine mediators secreted by immune and other cells, in response to infectious and certain malignant stimuli (1–3). IFNs have immune-modulating, antiviral and anti-proliferative properties which make them effective therapeutic agents for various medical conditions (4–7). Consequently, since their discovery, they have been studied extensively for their use in viral respiratory illnesses (8).

IFNs comprise three subfamily types; I, II, and III, classified depending on their sequence relatedness and surface receptor binding (1, 2, 4, 9). The largest subfamily type I IFNs includes IFN-α (leucocyte IFN) and IFN-β (immune IFN), which are secreted by virally-infected cells (9, 10). IFN-γ(immune IFN) is mainly secreted by natural killer and natural killer T-cells after antigen exposure. The type III subfamily includes IFN-λ which has three subtypes (9, 11–13).

IFNs have shown potent anti-viral activity against respiratory viruses *in vitro*, however this has not yet consistently translated into vivo anti-viral effects (2, 5–8, 10–13). The main pitfall of interferons are the associated systemic adverse effects experienced with intramuscular or subcutaneous administration. These include flu-like symptoms (fatigue, fever, myalgia and headaches), pulmonary symptoms, gastrointestinal symptoms, neurotoxicity, and depression (1, 14–16).

### Route and Dosing of IFN Administration

Interferons are poorly absorbed orally due to their large amino-acid sequence which is susceptible to digestive enzymes (17, 18). Effective absorption has been noted via intravenous, subcutaneous, intramuscular, and intranasal routes (17). Intravenous, subcutaneous, and intramuscular routes of administration can be associated with serious systemic side effects (5, 17, 18). Intranasal administration is associated with a more targeted effect on the upper airway which limits systemic adverse effects, however it can still result in local side effects such as mucosal irritation, drying, erosion and blood-stained mucus (5, 17, 18). These are dependent on treatment duration and dosing (5).

### The Link Between Respiratory Viruses and IFNs

Respiratory viruses such as influenza, respiratory syncytial virus (RSV), human metapneumovirus, parainfluenza, human rhinoviruses (HRV), human seasonal coronaviruses, and SARS-CoV-2 can lead to serious respiratory disease and mortality (19–21). Respiratory viral illness severity ranges from asymptomatic carriage, mild upper respiratory tract symptoms (common cold) to severe pneumonia, bronchiolitis and acute exacerbations of asthma or COPD which can be life-threatening (22). *In vitro* and animal studies have shown successful suppression of respiratory viruses on administration of exogenous IFNs (23–29). Consequently, IFNs have been investigated *in vivo*.

### Intranasal IFN-α for Prevention and Treatment of Colds Due to Rhinovirus

Generally, trials investigating either leukocyte-derived human interferon (HuIFN) or recombinant HuIFN-α2 (rHuIFN-α2) as prophylaxis against either experimental or natural rhinoviruses have been positive. Daily dosing of 10 million international units (MU) of rHuIFN-α2 or lyophilized HuIFN-α2 prevent rhinovirus colds and viral replication for both natural or experimentally induced infections (30–36).

Better prophylactic efficacy was noted with higher and or prolonged IFN-α dosing. Furthermore, administration a few hours before experimental rhinovirus infection inoculation conferred better symptom reduction (30, 33–36). High dose intranasal IFN-α2 was associated with increased local adverse effects such as nasal dryness, blood stained mucus and rhinoscopic findings of mucosal damage in most trials (30, 37–39). A trial using high-dose (10 vs. 20-MU vs. placebo) rHuIFN-α2 daily for 5 days was ineffective in treating naturally occurring RV colds. The 20-MU arm experienced prolonged duration of pronounced cold symptoms, more clinically significant adverse effects and secondary complications requiring antibiotic administration in comparison with the placebo and 10-MU groups (39). A study comparing two intranasal methods using a high-dose (9MU) HuIFN-α2 administered three-times daily, for 5 days, did not prevent the development of experimental RV colds. This study did however indicate that nasal drop administration was more effective than nasal spray in improving the clinical course and reducing the duration and quantity of viral shedding (40).

Two placebo-controlled double-blind family studies (performed in America and Australia) assessing the efficacy of a week of daily 5-MU IFN-α2 as post-exposure-prophylaxis against the common cold in exposed household contacts, showed a reduction in incidence of RV infections, but were generally ineffective for other viruses (37, 41). In contrast, a family study conducted in Seattle, using the same protocol, showed no reduction in the overall number of colds or secondary colds in family contacts who received IFN-α2. The authors suggested that the lack of preventive efficacy of IFN-α2 could be due to a higher prevalence of influenza B in the study location, Seattle, in comparison with the family studies performed in Virginia and Adelaide where RVs were more prevalent (42). Similarly, another family study, performed in Switzerland, using low-dose rHuIFN-αA (0.3 or 1.5-MU daily for 5 days) resulted in a statistically insignificant reduction in cold transmission, but appeared to almost halve the mean duration of illness (*p* = 0.07) in family contacts (43). Post-exposure prophylaxis for asymptomatic family contacts in a Michigan study using IFN-α2b (5-MU on day 1 and 2.5-MU daily for 4 days thereafter) was ineffective in preventing RV colds. The authors thus concluded that a minimum dose of 5-MU IFN-α2 in family contacts is necessary to achieve acceptable post-exposure RV protection (44). These findings suggest that it is possible for high-dose IFN-α2 to reduce the spread of common colds in family settings, particularly in locations where RV infections are prevalent (37, 41–43).

### Intranasal IFN-α as Protection Against Other Respiratory Viruses

IFN-α administration as prophylaxis against other respiratory viruses has shown varying results. Intranasal prophylactic IFN-α administration in experimental coronavirus, RSV and influenza A has shown reduction in virus yield, infection frequency and symptom scores (45–47).

A study using HuIFN-α prophylactically, a day before influenza B inoculation, slightly delayed the onset of infection but did not prevent illness or reduce its severity (31). Assessment of IFN-α2b as seasonal prophylaxis for respiratory infections, demonstrated a significant reduction in the number of rhinovirus, but not parainfluenza, infections (38).

### Studies Using IFN-β

Type I-β IFNs have been trialed in the hope that they might have better tolerability and efficacy. There is conflicting evidence regarding the efficacy of recombinant IFN-β-serine (rIFN-βser) as prophylaxis against experimentally induced rhinovirus colds. RIFN-βser has demonstrated *in vitro* anti-viral activity against both rhinoviruses and coronaviruses (38, 48). Most trials using intranasal rIFN-βser have shown efficacy in preventing common colds and are associated with fewer local adverse effects than IFN-α (48, 49).

In contrast, two RCTs in 1986 and 1987 demonstrated rIFN-βser nasal drops to be ineffective as natural cold prophylaxis, even at a higher doses, which were associated with limited local adverse effects (50).

### Studies Using Type II IFN-γ

Two studies assessing the effectiveness of rHuIFN-γ as prophylaxis against experimental RV showed no benefit and were associated with symptom worsening and a high rate of local side effects (51).

### Type III IFN-λ

IFN-λ was discovered later than the other two types and it plays a key role in respiratory viral infections. It is induced earlier than type I IFNs, mounting an immune response which can overcome viral infection when viral load is low (52). *In-vitro* work has confirmed that IFN-λ is the primary IFN produced by bronchial epithelial cells during the innate response to viral infections (53).

Not only does the specificity of the response to viral respiratory infections with IFN-λ highlight its therapeutic potential, but it may also bring a more favorable side effect profile. In mice, nasal IFN-λ demonstrated a superior anti-influenza therapeutic effect, reducing viral load and crucially not inducing the same pro-inflammatory effect as its comparator, IFN-α (54). Allowing for favorable translation between *in-vitro* and *in-vivo*, murine and human, and subcutaneous and nasal, IFN-λ presents the most promising therapeutic profile of all IFNs. To date, no nasal IFN-λ studies have been identified.

### IFNs and Chronic Respiratory Conditions

#### Asthma

Asthma sufferers of all ages frequently have impaired antiviral immunity. Several studies demonstrate deficient IFN-β and IFN-λ induction after viral infection of primary bronchial epithelial cells (pBECs) as well as deficient induction of IFN-α, IFN-β, and IFN-λ by viral infection of macrophages/dendritic cells (55–60). IFN deficiency may explain virally induced acute asthma exacerbations (55–60) since IFNs suppress viral replication in pBECs (61).

A trial of inhaled IFN-β on asthma symptoms due to viral infections did not show significant improvement of asthma symptoms in the whole study population (62). There were improved morning peak flows, reduction in additional treatment required and enhanced innate immunity as evidenced by serum and sputum biomarkers. A subgroup analysis of moderate/severe asthma did show significant improvement in symptoms and indicated a need for larger trials (62). According to an abstract, a trial investigating SNG001 (inhaled IFN-β) for the prevention or reduction of asthma symptoms after the onset of a respiratory viral infection, have shown that SNG001 maintained antiviral response during the treatment period (63).

Another abstract of a larger trial assessing the use of on-demand SNG001, for treating asthmatic patients with upper-respiratory-tract infection symptoms, did not meet its primary end-point of a reduction of severe exacerbations due a lower than expected number of virally induced severe exacerbations. It demonstrated improved morning peak expiratory flow readings during days 1 to 7 of the treatment period (64).

#### Chronic Obstructive Pulmonary Disease

Similarly to the IFN deficiency in pBECs of asthmatics, COPD studies have shown impaired virus-induced IFN production in pBECs and bronchoalveolar lavage (BAL) cells (65, 66). This supports a causal relationship between rhinovirus infections and acute exacerbations of COPD (66). A press release for a phase 2 clinical trial investigating inhaled SNG001 in COPD patients with a confirmed respiratory viral infection, has confirmed SNG001 was well-tolerated with enhanced lung anti-viral responses in comparison with placebo. Furthermore, patients already requiring oral corticosteroids and or antibiotics at the time of randomization into placebo or treatment (SNG001) groups demonstrated significantly better lung function during the treatment period (67).

### Recent Trials Using IFNs for Treating COVID-19

#### Nasal IFNs

SARS-CoV-2 is a zoonotic, enveloped positive- stranded RNA coronavirus first identified in December 2019, now causing a global pandemic. The well-established antiviral properties of IFN have attracted interest in this context. Nasal administration might inhibit SARS-CoV-2 in the nasal epithelium, as well as bolstering the nasal immune barrier (68, 69).

Conversely, *in-vitro* work has implicated viral-driven IFN inflammation in upregulation of ACE2 receptors, the SARS-CoV-2 binding receptor, in nasal epithelial cells (70). Inter-individual IFN responsiveness has been postulated as one factor among a host of others which might explain inter-individual variability in COVID-19 severity. This has led to the hypothesis that blockade of the IFN effect might reduce disease severity (71).

One of the earliest nasal IFN trials was an open-label trial of 2,941 Chinese healthcare workers in one hospital who were split into two groups: those working in areas conferring a high risk of exposure to SARS-CoV-2 and those working in low- risk areas (69). The low- risk group took 2–3 drops of 3,000 μg/mL rhIFN-α in each nostril four times a day for 28 days, and the high-risk group took the nasal drops with an additional weekly subcutaneous injection of 1.6 mg thymosin-α-1, a polypeptide hormone which mediates T-cell response, for 28 days. No new COVID-19 infections were identified by 28 days after therapy ended, which the authors contrast to a “control group” of healthcare workers infected regionally and nationally in other studies spanning the same time frame. The 0% infection rate is striking, and the adverse event profile is promising, limited to “a few… participants experienc[ing] transient irritation of the nasal mucosa,” but comes with serious caveats. This trial was not blinded, randomized or conducted with a comparable control arm. The majority (around 80%) of participants were in the low-risk group, and all were reportedly strictly adherent to personal protective equipment guidance, including enhanced PPE measures in the high-risk group. Efficacy of nasal IFN administration against SARS-CoV-2 infection is not substantiated by this preprint alone (69).

Reports from Cuba have described the prophylactic use of intranasal recombinant human IFN-α2b (marketed as Nasalferon) in both asymptomatic travelers arriving at airports and healthcare staff. Very limited information is available, save for a short letter outlining the adverse event profile of the twice daily preparation in 420 participants: 17.4% reported headaches and 3.1% reported weakness. All participants had a negative PCR result at enrolment and no participants were infected at 15 days (defined by examination and PCR result) (72).

#### Subcutaneous IFNs

There are several studies assessing the efficacy of subcutaneous (SC) IFNs in COVID-19 disease and one of nebulised IFN. A randomized controlled trial used subcutaneous IFN-β1b in conjunction with lopinavir-ritonavir and ribavirin within 7 days of infection (against lopinavir-ritonavir alone), finding that viral shedding, hospital stay and severity of patient observations were markedly reduced, although there was no mortality across groups and insufficient numbers to assess other clinical endpoints (73).

A double-blind RCT of a single dose (180 mg) of SC pegylated IFN-λ (peginterferon-λ) on outpatients with laboratory-confirmed mild to moderate COVID-19 showed an accelerated decline in the SARS-CoV-2 virus, with a high proportion of patients clearing the virus by day 7, in the IFN group in comparison with the placebo (74). In contrast, another trial, on outpatients with mild to moderate COVID-19 disease, using the same dose but SC peginterferon-λ-1a, did not show reduction in the duration of SARS-CoV-2 viral shedding or an improvement in symptoms in comparison with placebo. This result could have been due to the later administration of the peginterferon at a median symptom duration of 5 days (at randomization) with 40% of participants already having positive SARS-CoV-2 IgG results at enrolment; whereas the former trial by Feld et al. had a median time from symptom onset of 4.5 days (SD 1.7) alluding to slightly earlier administration of peginterferon (75). An RCT, in Iran, assessing SC IFN-β1a (12M IU) in the treatment of severe COVID-19 in addition to SOC (standard of care treatment: hydroxychloroquine plus lopinavir-ritonavir or atazanavir-ritonavir) in comparison with SOC alone, demonstrated a reduction in 28-day mortality (PMID: 32661006), with early IFN administration significantly and markedly reducing mortality (76). A second RCT, this time open-label, performed in Iran, assessing the efficacy and safety of longer-term IFN-β1b (250 mg SC every other day for 2 weeks) in the treatment of severe COVID-19 reported a shorter time to clinical improvement (*p* = 0.002), more discharged patients at day 14 (*p* = 0.03) and reduced ICU admission rates (*p* = 0.04) (77).

Another small three-armed study of SC IFN-β1a (12 000 IU on days 1,3,6) and IFN-β1b (8M IU on days 1, 3, 6), comparing them against each other and a control group, reported shorter time to clinical improvement with IFNβ1a against the control group (HR; 2.36, 95% CI 1.10–5.17, *P* = 0.031) while IFNβ1b had no significant difference compared with control; HR; 1.42, (95% CI 0.63–3.16, *P* = 0.395). The median time to clinical improvement for both of the intervention groups was 5 vs. 7 days for the control group. The mortality was numerically lower in both of the intervention groups (20% in the IFNβ1a group and 30% in the IFNβ1b group vs. 45% in the control group) (78).

Addition of SC pegylated IFN α-2b (PEG IFN-α2b) in moderate COVID-19 to SOC did better than SOC alone. Results showed that 19 (95%) subjects in PEG IFN-α2b plus SOC group had achieved clinical improvement on day 15 compared to 13 (68.42%) subjects in the SOC group (*p* < 0.05). Overall, 80 and 95% of subjects in the PEG IFN-α2b plus SOC group had a negative RT-PCR result on day 7 and day 14, respectively, compared to 63 and 68% in the SOC group (79).

The World Health Organisation's large Solidarity trial randomly assigned patients equally to one of four antiviral drugs (remdesivir, hydroxychloroquine, lopinavir, and IFN-β1a) in comparison with control drugs, in hospitalized patients with COVID-19. The IFN-β1a group either received local standard of care (SOC) or lopinavir with ritonavir plus IFN-β1a [three doses 44 micrograms of SC (and in some cases 10 microgram IV doses daily for 6 days when patients were on high-flow oxygen, ventilation or extra-corporeal membrane oxygenation)] IFN-β1a (on the day of randomization, day 3 as well as 6,). Results showed that none of the drugs reduced mortality, initiation of ventilation or duration of hospitalization (80).

#### Nebulized IFNs

A genetically engineered super IFN-α (rSIFN-co) administered via nebuliser showed a better outcome than regular IFN-α, in a randomized (1:1) trial, in patients hospitalized with moderate-to-severe COVID-19 who received either nebulised rSIFN-co or IFN-α nebulization added to baseline antiviral agents for no more than 28 days. Time to clinical improvement was 11.5 vs. 14.0 days (95% CI 1.10–2.81, p = 0.019); the overall rate of clinical improvement on day 28 was 93.5 vs. 77.1% (difference, 16.4%; 95% CI 3–30%); the time to radiological improvement was 8.0 vs. 10.0 days (*p* = 0.002), the time to virus nucleic acid negative conversion was 7.0 vs. 10.0 days (*p* = 0.018) (81).

Nebulised IFN-α2b was assessed in an uncontrolled exploratory study alone or in combination with arbidol hydrochloride (an antiviral with immune enhancing activity), performed on 53 SARS-CoV-2 PCR positive patients. This showed an apparent shorter duration of viral shedding and a reduction in acute inflammatory markers such as CRP and IL-6 (82).

Another study using IFN-α2b via spray inhalation to 68 patients matched with 36 case controls, with both groups with PCR confirmed COVID-19, did not demonstrate reduced viral shedding but signaled shorter hospitalization times (83).

A randomized controlled trial using inhaled IFN-β-1a showed that in patients with COVID-19 the odds ratio of developing severe disease was 0.28 in the treatment arm compared with placebo (*p* = 0.043). Improvement in the chance of recovery during the treatment period and in symptoms were also demonstrated (84).

Finally, a randomized, open-label parallel group trial of inhaled aerolised Novaferon (a novel interferon manufactured from recombinant antiviral protein) and Novaferon plus Lopinavir/Ritonavir groups demonstrated significantly higher viral clearance rates on day 6 than the Lopinavir/Ritonavir group (50.0 vs. 24.1%, *p* = 0.0400, and 60.0 vs. 24.1%, *p* = 0.0053). The median time to viral clearance was 6, 6, and 9 days (85).

Overall, in COVID-19 disease, there is reasonable biological plausibility for the use of nasal IFN therapy, although as yet very little evidence to support its use. RCTs would be needed to demonstrate both efficacy and acceptable side effect profiles, the latter a factor suggesting that this therapy may be best suited to individuals who are facing the prospect of a high risk of infection (e.g., international air travel, working with symptomatic patients with COVID-19, attending crowded public events). There is however substantial evidence supporting the use of SC IFN therapy in mild to moderate COVID-19 disease, with more benefits evident when administered early. Studies should investigate the use of nasal IFN therapy as both prophylactic therapy and an early intervention to prevent progression. In addition use of nasal IFN might prevent the recently described and nasal colonization by SARS-CoV-2 in patients with prolonged anosmia (86).

#### The Future of IFNs

IFN therapy is an extremely promising, and in some areas proven treatment for respiratory viral infections and their sequelae. Nasal IFNs bring many of the same benefits without the systemic side effects which are occasionally poorly tolerated by patients. Localized side effects of nasal IFN persist and require strategies for minimization. The therapeutic potential of IFN is pertinent amidst the current global COVID-19 pandemic. Since inhaled IFN-β is well-tolerated and effective in the lung, it is likely that intranasal treatment would also be well-tolerated and effective. IFN-λ is likely to be even better tolerated and trials of intranasal IFN-λ are eagerly anticipated.

## Intranasal Probiotic Therapies for Rhinitis and Rhinosinusitis

Over the past decade, there has been an increase in understanding of the importance of bacterial communities present on all body surfaces and cavities (87). These bacterial communities, consisting of trillions of individual bacteria from different species and their genomes, are collectively termed the “microbiome.” The term “microbiota,” referring only to the microbial taxa associated with humans, should not be used interchangeably with “microbiome.” Every surface and cavity of the body has a specific microbiome which can vary dramatically between individuals, for instance, the hand or gut microbiome can be 80–90% different between individuals (88, 89).

The human microbiota consists of 10–100 trillion microbes, primary harbored in the gut (90). In fact, much of what we know about microbe-host interactions and associations between dysbiosis and disease states stems from the gut microbiome. Diversity of the gut microbiome is emerging as a critical determinant of host health, and a loss of diversity has been associated with a variety of gastrointestinal and systemic diseases (91–94) including allergy (95). Dysbiosis is a loosely defined concept referring to any change in the microbiome that adversely affects the health of the host organism. Dysbiosis can be characterized by broad shifts in community microbial compositional structures, reduced species diversity, and changes in the relative proportion of organisms, whereby there is relative lack of “health-associated” bacteria. “Healthy” bacteria are associated with regulation of immune responses, defense against pathogenetic bacteria, and epithelial regeneration or repair of epithelial surfaces (96).

The nasal microbiome has been linked to several immune system disorders and infectious diseases such as allergic rhinitis (AR), chronic rhinosinusitis (CRS), acute respiratory tract infections (ARTI), otitis media (OM), and asthma. Previously, the persistence of pathogenic bacteria in the nasal cavity was believed to cause disease such as the overabundance of *Staphylococcus aureus* producing superantigens and toxins, impairing immune detection and activation, and ultimately damaging the fragile respiratory epithelium (97, 98). From the perspective of the microbiome, disease can be associated with an imbalance between the commensal microbiome and bacterial pathogens, resulting in a reduction in commensal bacterial diversity, combined with an increase in the growth of microbiomes eliciting an inflammatory response resulting in symptoms of rhinitis. The goal of this review is to contextualize the use of probiotics for the sinus, specifically for AR and CRS, with a focus on pre-clinical studies, due to limited data on the intranasal probiotic formulations in humans.

### Dysbiosis and Allergic Disease

AR is an inflammatory disease of the nasal mucosa, triggered by allergen exposure. AR is common and previously estimated to affect 10–30% of the population worldwide (99). A potential role for microbial exposure in allergy risk was identified in the late 1980s with the observation that children from larger households tended to have lower rates of AR and eczema (100). This contributed to the hygiene hypothesis relationship which postulates that a reduction in the frequency of infections, due to reduced exposure to microorganisms, is associated with an increase in the frequency of allergic diseases (100). This hypothesis is supported by robust epidemiological data (100–103). A notable example highlighting the importance of the interaction between the environment, host microbiome, and allergy comes from a comparison of genetically similar populations of Eastern and Western Europe (104, 105). The gut microbiota of infants from Eastern Europe, where the prevalence of atopy is low, and Western Europe, where it is high, have been reported to be distinct (104, 105). Consistent with the sequence of the atopic march, the gut microbiome composition of children with food sensitization from both Western and Eastern Europe has also been found to be distinct from those without atopic diseases from these geographical regions (104, 105). There is even some evidence suggesting a relationship exists with the nasal microbiome, specifically. Ruokolainen et al. examined the prevalence of allergic diseases and both skin and nasal microbiota in 180 children, ages 7 to 11, from Finnish and Russian Karelia. These regions have relatively identical climatic and geographic features, except Russian Karelia is mainly a rural environment and Finnish Karelia is a modernized area. AR, atopic eczema, atopic sensitization, asthma, and self-reported rhinitis were 3- to 10-fold more common in children from Finnish Karelia. Moreover, the nasal microbiome was significantly more diverse among Russian participants than Finnish subjects (106).

A few studies characterize the nasal microbiome in AR, with conflicting results. A 2014 study reported increased bacterial diversity in the middle meatus of seasonal AR participants compared to healthy controls (107). However, these results could not be replicated in a study by Lal et al. (108). More recently, Hyun et al. (109) demonstrated that dysbiosis of the inferior turbinate was associated with high levels of total IgE but not AR occurrence. High levels of total IgE in AR patients were linked to an increased *Staphylococcus aureus* population and decreased *Propionibacterium acnes* in the nose. Dysbiosis of the nasal microbiota was not associated with the number of sensitized allergens or individual allergen specific-IgE levels (109). More studies are desperately needed in this area, especially within the context of a validated disease model such as a controlled allergen challenge facility.

### Dysbiosis and Chronic Rhinosinusitis

CRS is considered an inflammatory disease of the nasal and sinus cavities with sinonasal symptoms lasting for 12 weeks or more (110). CRS affects ~3 to 5% of the Canadian and 12% US populations respectively (111, 112). Several risk factors have been associated with the development of CRS including smoking, lower income, and a history of allergy, asthma, or chronic obstructive pulmonary disease (COPD) (111). Reputed pathological factors include changes in the microbiota, imbalance of the local or systemic immune system, allergens, toxins and genetic pre-disposition (113–116). A 2016 meta-analysis (117) of studies comparing the composition of the bacterial nasal microbiome in CRS patients compared to healthy controls found reduced diversity and less stable bacterial networks in CRS patients (118). These findings have been supported in more recent studies (119, 120). No consistent patterns of one specific microbiome has been observed in all CRS patients, although, previous descriptive studies have shown that the nasal microbiome most frequently includes coagulase-negative *Staphylococcus, Pseudomonas aeruginosa*, and *S. aureus*. Importantly, the nasal microbiomes of CRS patients with and without nasal polyps are different in comparison to healthy individuals (108, 121) suggesting the nasal microbiota profile may modulate CRS phenotype (117).

### Therapeutic Manipulation of the Microbiome

Taken together, these findings suggest the possibility of improving health by modifying the microbiome to a desirable composition or functional state rather than elimination of the pathogenic bacteria. Perhaps the first example of microbiome supplementation therapy is fecal microbiota transplantation (FMT). FMT involves transferring communities of microbes from a donor to a recipient. Thus far, FMT has been most notably used for treating *Clostridium difficile* colitis, where fecal material from healthy donors is transplanted to patients with the disease (122, 123). Despite the promising results of FMT to treat this condition, several barriers remain with directly transferring live bacteria between humans (124). Excitingly, a small Phase I open-label trial to evaluate the safety and tolerability of oral encapsulated FMT administered open-label over 2 days for the treatment of peanut allergy in 10 adult subjects is currently underway (Clinicaltrial.gov identifier: NCT02960074).

Probiotics are defined as “live microorganisms that, when administered in adequate amounts, confer a health benefit on the host” (125). The most common microorganisms used as probiotics are from the *Bifidobacterium* and *Lactobacillus* genera which are the predominant and subdominant groups of the gut microbiome, respectively (126). Probiotics exert their beneficial effects by modulating inflammation, secreting small molecules which may act at a distance, and restricting pathogenic bacterial growth via direct inhibition and competition for scarce nutrients. Certain strains have beneficial effects on epithelial regeneration and repair (127). Several probiotic strains, such as *Lactobacillus rhamnosus GG, Streptococcus thermophiles, Lactobacillus plantarum MB452*, and the gram-negative probiotic strain *Escherichia coli Nissle* 1917 has been shown to increase the epithelial barrier integrity of tight junction- related genes or adherent junction- related genes (128–131). Probiotics interact via their microorganism-associated molecular patterns, with pattern recognition receptors on epithelial cells. This interaction can regulate tight junctions and adherence junctions, which can result in the restoration of epithelial barrier integrity (132). It is important to stress that the biological effects of probiotics are strain specific and therefore, it is vital to use isolates with documented probiotic properties.

Probiotic treatments may be clinically beneficial for individuals suffering from AR, particularly in combination with perennial AR treatment. The literature is well-summarized in a systematic review of probiotics in AR's treatment by Güvenç et al. (133) who concluded that significant evidence suggests beneficial clinical and immunologic effects of probiotics. A caveat is that the probiotic clinical trials evaluated by this review relied on oral dosing, and research into direct nasal probiotics is scarce. Likewise, probiotics treatments have been suggested as an intervention option for CRS; however, the current literature has not supported this concept. One reason for this lack of beneficial effects in CRS could be diverse endotypes and phenotypes in CRS. The pre-clinical and clinical trials described herein support the use of nasal probiotic formulations in AR and CRS.

### Probiotics for Sinonasal Disease

Oral administration of *Lactobacillus rhamnosus* GG was previously shown to offer benefits in the context of allergic disease prevention and treatment, both in animal models (134, 135) and in human clinical trials (136–138). Intranasal application of the live probiotic *Lactobacillus rhamnosus* GG bacteria can decrease allergic airway inflammation and lung Th2 cytokine production, and is even capable of preventing airway hyperactivityinduced by repeated intranasal application of birch pollen extract in mice (139). Repa et al. tested the capacity of two lactic acid bacteria (LAB) strains, *Lactococcus lactis* MG1363 (*L. lactis*, a dairy strain) and *Lactobacillus plantarum* NCIMB8826 (*L. plantarum*, a human isolate), to prevent or modulate allergic immune responses. The authors demonstrated that mucosal administration of the two LAB strains—*L. lactis* or *L. plantarum*—applied together with a birch allergen prior or after sensitization, induced a shift toward Th1 immune responses along with a reduction of Th2 dependent basophil degranulation (140). In a follow-up study, Daniel et al. used Bet v 1-producing LAB strains for mucosal prophylaxis in a mouse model of birch pollen allergy (141). They saw reduced allergen-specific IgE concomitant with increased allergen-specific IgA at the mucosae in mice. This suggests mucosal delivery of innocuous recombinant LAB may induce protective immune responses at the site of direct allergen exposure and may represent effective strategy in primary prevention of type I allergy (141). Positive results have also been seen in the context of food allergy. Intranasal administration of recombinant *Lactococcus lactis* strains expressing bovine β-lactoglobulin (BLG), a major cow's milk allergen, has been shown to partially prevent mice from sensitization (142) and when combined with interleukin-12 producing *L. lactis* to inhibit the allergic reaction to BLG (143).

In CRS patients and a subsequent mouse model of sinusitis, Abreu et al. found an increase in the relative abundance of a single species, *Corynebacterium tuberculostearicum* compared to healthy controls. Further, this group found that intranasal inoculation with *Lactobacillus sakei* protected the sinus epithelium, putatively through competitive inhibition of *C. tuberculostearicum*, and may represent a novel therapeutic option for amelioration or prevention of sinus pathology, even in patients with severe sinus microbiome depletion (144).

Besides allergy and CRS, alternative uses for intranasal probiotic therapies have been investigated in animal models and should be noted here. In a neonatal model of influenza virus infection, intranasal application of *Lactobacillus rhamnosus GG* prior to influenza infection dramatically improves survival and provides and early increase in transcription of type I IFNs. The probiotic-related protection is MyD88-dependent and specifically involves TLR4 recognition of LGG (145). As mentioned previously, a major mechanism of action of probiotics is competitive exclusion of pathogens. Following these principles, the intranasal application of *S. epidermidis* has been shown to prevent colonization by methicillin-resistant *Staphylococcus aureus* in mice (146).

Oral probiotic treatments have shown some promise in humans, though this is not the case for CRS. Nasal probiotic formulations may be a more effective drug delivery approach for rhinitis particularly the *Streptococcus spp*., and *Lactic acid bacteria (LAB)*, highlighted in Table 1.

**Table 1:** Human clinical trials investigating intranasal probiotic formulations.

**Probiotic type**	**Probiotic strains**	**Disease**	**Treatment regimen**	**Population**	**Results**	**Author**	**Notes on formulation**
Alpha-haemolytic streptococci (AHS)	*S. sanguis*, *S. mitis*, and *S. oralis*	Acute otitis media (AOM)	One 50 μl puff each nostril OD for 4 months	43 children ≤ 4 y.o with AOM	No sig. dif. in episodes of AOM than placebo No sig. dif. in nasopharyngeal flora than placebo ↓*H. influenzae* in the active group	Tano et al. (147)	≥10^7^ CFU/ml in a suspension of 10% skim milk and 0.9% NaCl
	*S. salivarius 24SMBc*	Healthy adults	Two puffs per QID day at intervals of 4 h One puff: 8 x 10^9^ CFU/ml	20 adults ≥ 18 y.o	*S. salivarius 24SMBc* colonized the rhinopharynx tissues in 95% of subjects *S. salivarius 24SMBc* persisted in 55% of colonizers 6+ days from last dose (at 10^5^ CFU/ml)	Santagati et al. (136)	5 × 10^9^ CFU/mL in a water solution with dimethicone, without gas
	*S. sanguinis 89a*, or *L. rhamnosus* (LB21, NCIMB 40564)	Secretory otitis media (SOM)	Two 50 μl puffs per nostril BID for 10 days before trympanostomy tube surgery	60 children 1–8 y.o with SOM and 19 healthy controls	More patients treated with *S. sanguinis* (37%) were cured or much better after clinical recovery than *L. rhamnosus* (6%) or placebo (17%)	Skovbjerg et al. (137)	5 × 10^9^ CFU/ml in skim milk 0.9% NaCl
	*S. salivarius 24SMBc* and *S. oralis 89a*	Healthy adults	Two puffs per nostril 1 week	20 adults ≥ 18 y.o	↓ in *S. aureus* and other potentially harmful bacteria	De Grandi et al. (138)	*S. salivarius 24SMBc* and *S. oralis 89a* in a 98:2 ratio suspended in a PEG/PPG copolymer and pH 7.00-buffered isotonic solution
Lactic acid bacteria (LAB)	9 *Lactobacillus* spp. and 4 *Bifidobacterium* spp.	Healthy adults	One 100 μL puff to each nostril Single administration	22 adults ≥ 18 y.o	No adverse events (AE) or symptoms No sig. dif. in microflora No inflammatory response	Mårtensson et al. (139)	Spp. obtained from the honeybee Apis mellifera 1 × 10^11^ CFU/ml in water
	9 *Lactobacillus* spp. and 4 *Bifidobacterium* spp.	CRS	One 100μl puff per nostril BID for 2 weeks (1-week treatment, 1-week sham)	21 adults ≥18 y.o with CRSsNP	No AE or symptoms No sig. dif. in microflora No inflammatory response	Mårtensson et al. (140)	Spp. obtained from the honeybee Apis mellifera 1 × 10^11^ CFU/ml in water
	*Lactobacillus casei* AMBR2	Healthy adults	One puff BID for 2 weeks	20 adults ≥18 y.o	*L. casei AMBR2* colonized the nasopharynx in 60–95% of subjects for ≥10–16H after last dose	De Boeck et al. (141)	Spray-dried powder resolved in water One puff: 10^8^ CFU/ml
	*Lactococcus lactis W136*	CRS	One sinus irrigation BID for 2 weeks	24 adults ≥ 18 y.o with CRS refractory to previous medical and surgical therapy	Improvements in symptoms, measures of quality of life, and the mucosal aspect as assessed by endoscopy ↑*D. pigrum and ↓ S. aureus* and ↓ P. aeruginosa	Endam et al. (142)	1.2 × 10^9^ CFU/ml in buffered 0.9% NaCl One sachet: 1.2 × 10^9^ CFU/ml

*S. salivarius* and *S. oralis* are alpha-hemolytic streptococci (AHS) isolated from the human pharynx. Together they represent the predominant species in the upper respiratory healthy flora and have been shown to selectively influence the microbiota. Studies in otitis media patients have vetted intranasal administration of *S. salivarius* and *S. oralis* proving it is safe, well-tolerated and able to reduce the risk of acute otitis media in otitis-prone children (147, 148). Whether intranasal administration of AHS is effective as a treatment for otitis media remains controversial (147, 149).

Recently, the ability of *S. salivarius* and *S. oralis* to colonize and modulate the nasal microbiome has been investigated. De Grandi et al. investigated the effects of a 7-day treatment regimen of *S. salivarius* 24SMBc and *S. oralis* 89a in 22 healthy volunteers. After treatment, they a saw significant temporary decrease in *Corynebacterium diphtheriae, Haemophilus parainfluenzae, Moraxella catarrhalis, Prevotella denticola, Prevotella melaninogenica, Rothia dentocariosa, Staphylococcus aureus*, and *Streptococcus pseudopneumoniae*. These findings suggest a potential ability of *S. salivarius* 24SMBc and *S. oralis* 89a to regulate and reorganize the nasal microbiota composition, possibly favoring those microorganisms that may be able to limit the overgrowth of potential pathogens (150).

As described above, several studies investigating intranasal formulations of LAB have produced positive results in murine models. Furthermore, LAB are enriched in the healthy human nose and nasopharynx. In 2016, Mårtensson et al. examined the safety profile of delivering honeybee lactic acid bacteria (HLAB) directly into the nasal passage, using a spray. The administration did not produce any symptoms, or change inflammatory biomarkers of the nasal cavity, and did not alter commensal bacteria (151). The same group repeated such administrations in patients with CRS with nasal polyps for 2 weeks. Treatment was well-tolerated but did not reduce nasal symptom severity or inflammatory markers (152).

Another promising LAB includes *Lactobacillus casei* AMBR2, whose safety for intranasal application in healthy humans was recently confirmed (153). Currently, a clinical trial is ongoing to deliver proof-of-concept that *L. casei* AMBR2 can colonize the upper respiratory tract of health volunteers and CRS patients after daily nasal application via a nasal spray for 2 weeks (ClinicalTrials.gov Identifier: NCT03587545).

Recently, Ednam et al. completed a prospective open-label pilot trial of safety and feasibility for *Lactococcus lactis* W136 and observed positive results. Twenty-four patients received 1.2 billion CFU of *L lactis* W136 self-applied directly to the nasal and sinus passages twice-daily for 14 days via nasal and sinus irrigation. Therapy was well-tolerated and led to improvements in symptoms, measures of quality of life, and improvement in the mucosa as assessed by endoscopy. Gene expression profiling to identify implicated mechanisms suggested enhanced epithelial repair and regeneration and modulation of inflammation. Microbiome profiling using 16s technology showed an increase in the beneficial bacteria *Dolosigranulum pigrum* and reduced in the pathogens *Staphylococcus aureus* and *Pseudomonas aeruginosa* (154).

Nasal probiotic formulations may be a more effective drug delivery approach for allergic disease (Table 1); however, more studies are needed in this area. Future studies should investigate using a combination of nasal probiotics and immunotherapy to improve pre-existing treatments.

Potential uses for intranasal probiotic therapy may extend to Coronavirus disease 2019 (COVID-19). Intranasal administration of probiotic *Lactococcus lactis* W136 is being investigated as a potential therapy for ambulatory SARS-CoV-2 infection (Clinicaltrial.gov identifier: NCT04458519). It is suspected that innate immune signaling via the TLR1/2/6 motifs present on the bacterial surface and the TLR3 motifs in the cytoplasm could induce interferon gamma production, leading to clearance of COVID-19 infection during its early phases and helping regulate subsequent inflammatory events.

## Nasal Immunization

### Infection

The nasal route has great potential for vaccination because of its simplicity, painlessness, and ease of administration. The follicle-associated lymphoid tissues in the nasal epithelium induce mucosal immune responses, such as local IgA, in addition to serum IgG. Mucosal IgA neutralizes measles virus and Streptococcus pneumoniae, preventing further infection. In addition intranasal immunization can result in cross-reactive antibodies, possibly capable of cross- protection, thus increasing vaccine efficiency (155).

While there are clearly significant practical advantages to a needle-free vaccine delivered by the intranasal route, there are also some important disadvantages (155). Vaccines need to induce a long-lasting innate and adaptive immune response; however, there are some significant challenges to nasal immunization, as summarized in Box 1. A number of delivery systems including those based on liposomes, nanoparticles, virus-like particles and emulsions have been developed to overcome some of these barriers, with varying degrees of success (156).

Box 1 **Challenges to nasal immunization [adapted from Yusuf and Kett (155)].**

**Exposure:**

- Dilution of nasal antigens by mucosal secretions
- Reduced bioavailability due to mucociliary clearance, encapsulation of nasal antigens in nasal mucosal gel and inefficient uptake of antigen across the nasal epithelial barrier
- Degradation of vaccine by local proteases and nucleases

**Immunostimulation**:

- Need for a relatively large dose to ensure adequate immune response, yet limited delivery volume (typically 100–200 μL)
- Requirement for adjuvants to enhance immunogenicity, which may cause toxicity
- Higher molecular weight compounds (typically above 1 kDa) cannot be delivered via the intranasal route (157)

Probably the greatest success story for intranasal vaccination is the live attenuated (cold-adapted) influenza vaccine (LAIV). In the USA and Europe, this is marketed as Fluenz/Flumist®, however a nasal LAIV has been in use for over 50 years in Russia/USSR (158). Epidemiological data and mathematical modeling indicate children are the main spreaders of influenza infection (159). As a result, the vaccination of children has proven to be a very effective means of interrupting transmission and achieving disease control. Indeed, in two countries (UK and Finland), annual vaccination of children now forms part of the national immunization programmes. The intranasal route facilitates in-school vaccine administration.

LAIV consists of cold-adapted live influenza strains, which replicate locally (mimicking natural immune exposure) in the upper respiratory tract resulting in a mild, subclinical self-limiting immune response. The cold-adaptation prevents viral replication in the lower respiratory tract. The route of administration for LAIV is particularly well-suited to use in children. Furthermore, data suggests that intranasal LAIV results in a higher level of protection in children than the injected alternative (158, 159). However, more recent data from USA has indicated a reduction in efficacy against seasonal H1N1 strains in children (160). Recent changes in the strains included in the vaccine appear to have restored a replicative fitness and a reasonable level of efficacy (161). Perhaps the most noteworthy research finding from these changes has been the realization that serum antibody titres and seroconversion rates are poor correlates of protection in LAIV-vaccinated children (162); this highlights the key differences between assessing mucosal immunity induced by local (intranasal) vaccines and systemic immunity induced by parenteral vaccination.

The intranasal route has also been explored in developing vaccines against SARS-CoV-2 which has caused the COVID-19 pandemic. A number of Phase 1 studies are underway (163). In addition, at least one dual intranasal vaccine for both influenza and COVID-19 has been developed and is being evaluated (164). If successful, these would offer a significant advantage facilitating global mass immunization.

### Allergy

In contrast, surprisingly little research has been undertaken assessing the potential for intranasal immunotherapy against allergic disease, particularly allergic rhinitis. Nasal immunotherapy was first investigated 40 years ago, using both native allergen extracts and soluble allergoids (165). Early data indicated both the potential for efficacy as well as low rate of systemic adverse events (166–169). However, research into this route of administration appears to have been largely superseded by immunotherapy via the subcutaneous (SCIT) or sublingual (SLIT) route. A 2006 review summarized 21 double-blind placebo-controlled studies of local nasal immunotherapy, and reported that in 19, the clinical efficacy was at least equivalent to that reported for SCIT (170). Only one head-to-head comparison seems to have been published: Giannarini and Maggi randomized 45 grass-sensitized patients to either no treatment or open-label immunotherapy using SCIT or via the local intranasal route. There was a high drop-out rate: 37 completed the study, and only 25 (11 for nasal immunotherapy, 7 for the other arms) were evaluated after 2 years. Both local nasal immunotherapy and SCIT resulted in a similar improvement in symptom scores (171).

One concern with nasal immunotherapy is the potential to induce hyperresponsiveness (as is the case following allergen challenge (172) rather than allergen hyporesponsiveness. Reassuringly, the published data has demonstrated that hyporesponsiveness of the nasal mucosa can be achieved following exposure to low levels of allergen (173). At least in animal models, local nasal administration of antigens can induce interleukin-10 release in a manner akin to that seen with conventional subcutaneous immunotherapy (174, 175). Interestingly, in the above-mentioned study comparing nasal immunotherapy to SCIT, a significant reduction in allergen-induced T-cell responses was seen in both active treatment arms, but only SCIT induced an increase in IgG antibody production (175) which more recent studies have suggested is of critical importance in the clinical response to immunotherapy for allergic rhinitis (176).

It is unclear as to why further studies into local nasal immunotherapy have not been undertaken. It has been suggested that there may have been compliance issues due to frequent local nasal reactions, and/or difficulties in controlling the actual dose of allergen administered (165). However, in the study by Giannarini and Maggi, drop-out rates were lowest in the nasal immunotherapy arm. Similarly, dry-powder devices were developed to facilitate dose administration (169) One can only speculate that the nasal route was superseded by SLIT, where local reactions may be less frequent and probably less bothersome.

Finally, there is one report in a murine model of egg allergy where nasal immunotherapy using a liposomal-based delivery system resulted in desensitization to allergen challenge via the oral route (176) While intranasal challenge with food allergens causes a local allergic response (177) it is possible that the intranasal route could also be used to induce a degree of desensitization; this has not to date been formally assessed, and one has to consider whether the intranasal route would be an alternative to oral immunotherapy.

## Topical Treatment for Allergen- and Rhinovirus-Induced Rhinitis With Antibodies Bispecific for Allergens and ICAM-1

Allergic sensitization and rhinovirus (RV) infections are major causes of rhinitis. We propose intranasal application of antibodies bispecific for allergens and Intercellular Adhesion Molecule 1 (ICAM-1) as topical treatment for both allergic and RV-induced rhinitis. The immobilization of allergen-specific antibodies with ICAM-1-specific antibodies on the nasal epithelium should prevent washing out of the therapeutic antibodies and thus provide sustained inhibition of allergen transmigration through the epithelial barrier and of consequent allergic inflammation in the nasal mucosa. Since the majority of RV strains use ICAM-1 as receptor it should be possible at the same time to block RV infections.

**Figure 1:**
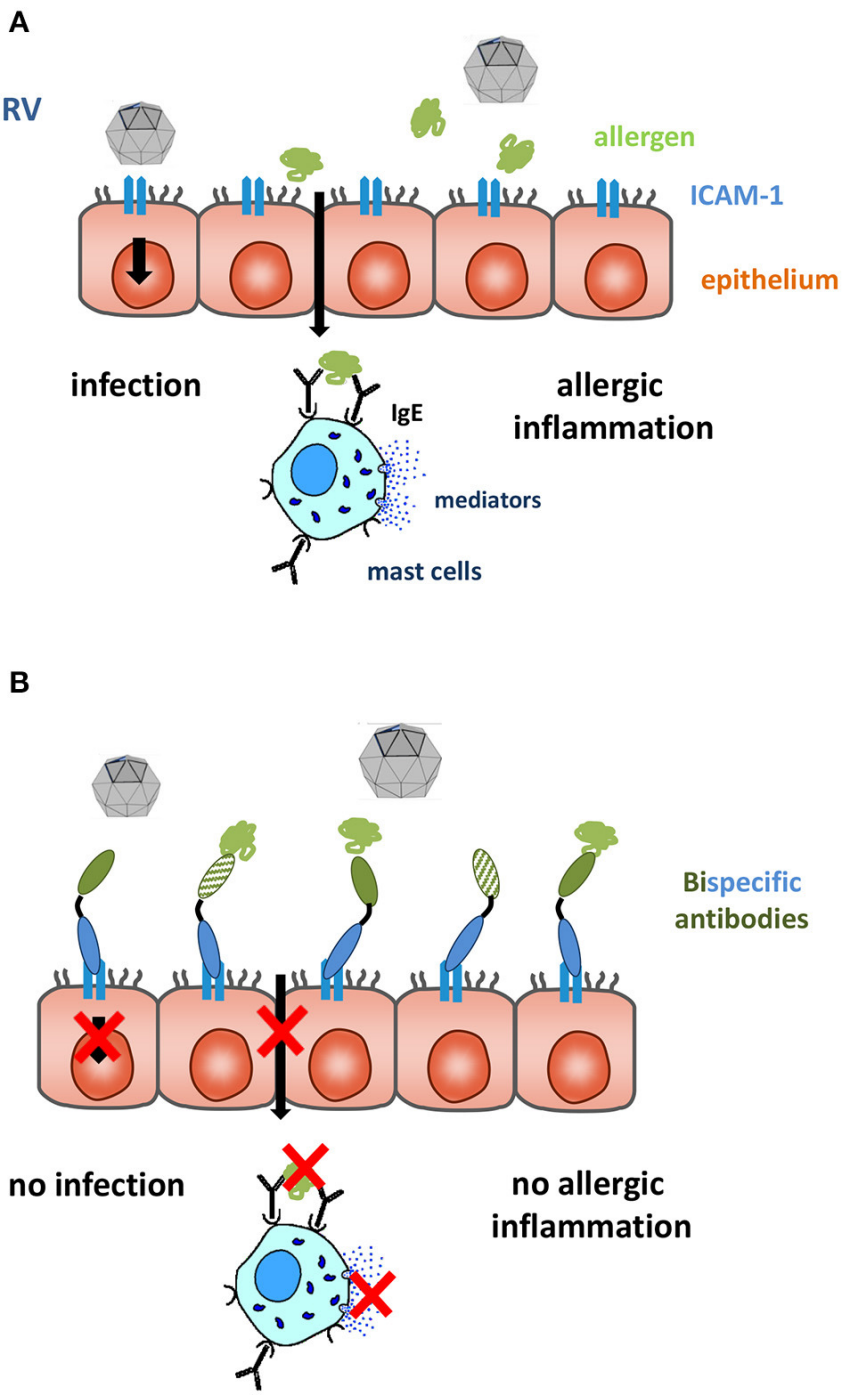
The respiratory epithelium is an important site of contact with allergens and RV. **(A)** Major group RVs infect the epithelium via their receptor ICAM-1 inducing tissue damage facilitating trans-epithelial penetration of allergens. **(B)** Bispecific antibodies binding simultaneously allergens (green) and ICAM-1 (blue) block RV binding to ICAM-1 and capture allergen. This may prevent RV- and allergen-induced inflammation. The immobilization of allergens on the apical side of the mucosa can be achieved by IgE blocking (*full green* domain) and IgE-non-blocking ***(****cross-hatched green* domain) allergen-specific antibodies, the epitope-specificity of the ICAM-1-specific antibody (full blue domain) decides if RV infections can also be blocked.

IgE-mediated allergy represents a common health problem affecting around one third of the world population (166). Allergic rhinitis is the most frequent manifestation of allergy. Rhinitis can be classified according to severity and appearance of symptoms as mild or moderate-severe and intermittent or persistent, respectively (178). Intermittent forms of allergic rhinitis are mainly caused by outdoor airborne allergens derived from pollen of grasses, trees and weeds (179–181). Allergic rhinitis is a major burden because it reduces the quality of life of affected individuals heavily (182). Among the non-allergic forms of rhinitis, virus-induced rhinitis, in particular rhinitis due to rhinovirus (RV) infections predominates (183). RV infections and allergen exposure in allergic patients trigger different pathways of inflammation. The majority of RVs infect the respiratory epithelium via binding to their receptor ICAM-1, minor group RVs bind to the low-density lipoprotein receptor (LDLR) and RV-C to cadherin-related family member 3 (CDHR3). Models for the first two virus-receptor interactions are available but RV-C is still difficult to isolate and propagate (184). RV-infections cause damage of the respiratory epithelium and local inflammation of the Th1 phenotype with production of inflammatory cytokines, leukocyte infiltrations and activation of the innate immune system (185). Allergens reaching IgE-sensitized mast cells after penetration of the respiratory epithelium of allergic subjects, cause immediate allergic inflammation by mast cell degranulation leading to release of biological mediators, cytokines and proteases and, upon chronic exposure also induce T cell- and eosinophil-mediate allergic inflammation (Figure 1A) (176).

Allergic sensitization, allergen exposure and RV infections can have synergistic effects in inducing rhinitis. For example, it is known that Th2 immunity impairs immune responses against RV infections which may render allergic subjects more sensitive to RV infections (186, 187). On the other hand, it has been shown that RV infections impair the barrier function of the respiratory epithelial cell layer and facilitate trans-epithelial penetration of allergens, thereby increasing submucosal allergen concentrations which potentially may aggravate allergic inflammation (188).

The use of antibodies specific for the binding site of RV on ICAM-1 has actually been considered as a possible approach for the treatment of RV infections (189). Regarding allergy, it is established that allergen-specific immunotherapy (AIT) induces allergen-specific IgG antibodies which compete with IgE for allergen binding and thus prevent allergic inflammation (190). The development of allergen-specific blocking IgG in serum is considered as a robust biomarker for success of AIT (191) and it has been shown that after AIT allergen-specific IgG increase also in nasal secretions where they can capture allergens (192). It is therefore reasonable to assume that it may be possible to combine local treatment approaches for rhinitis caused by RV infections and allergic inflammation by creating bispecific antibodies which bind to ICAM-1 and block RV infections and simultaneously capture allergens and prevent them from intruding through the respiratory mucosa as indicated in Figure 1B.

The feasibility of such an approach has actually been demonstrated by a series of *in vitro* experiments (193). In the coming paragraphs we will review the concept of allergen-specific blocking IgG in AIT in the context of technological advances made during the last decades regarding the production of human and in particular of human allergen-specific antibodies, which may now create the basis for a combined antibody-based topical treatment for allergen- and RV-induced rhinitis.

### Allergen-Specific IgG Antibodies Confer Protection Against Allergy: Historic Aspects

**Figure 2:**
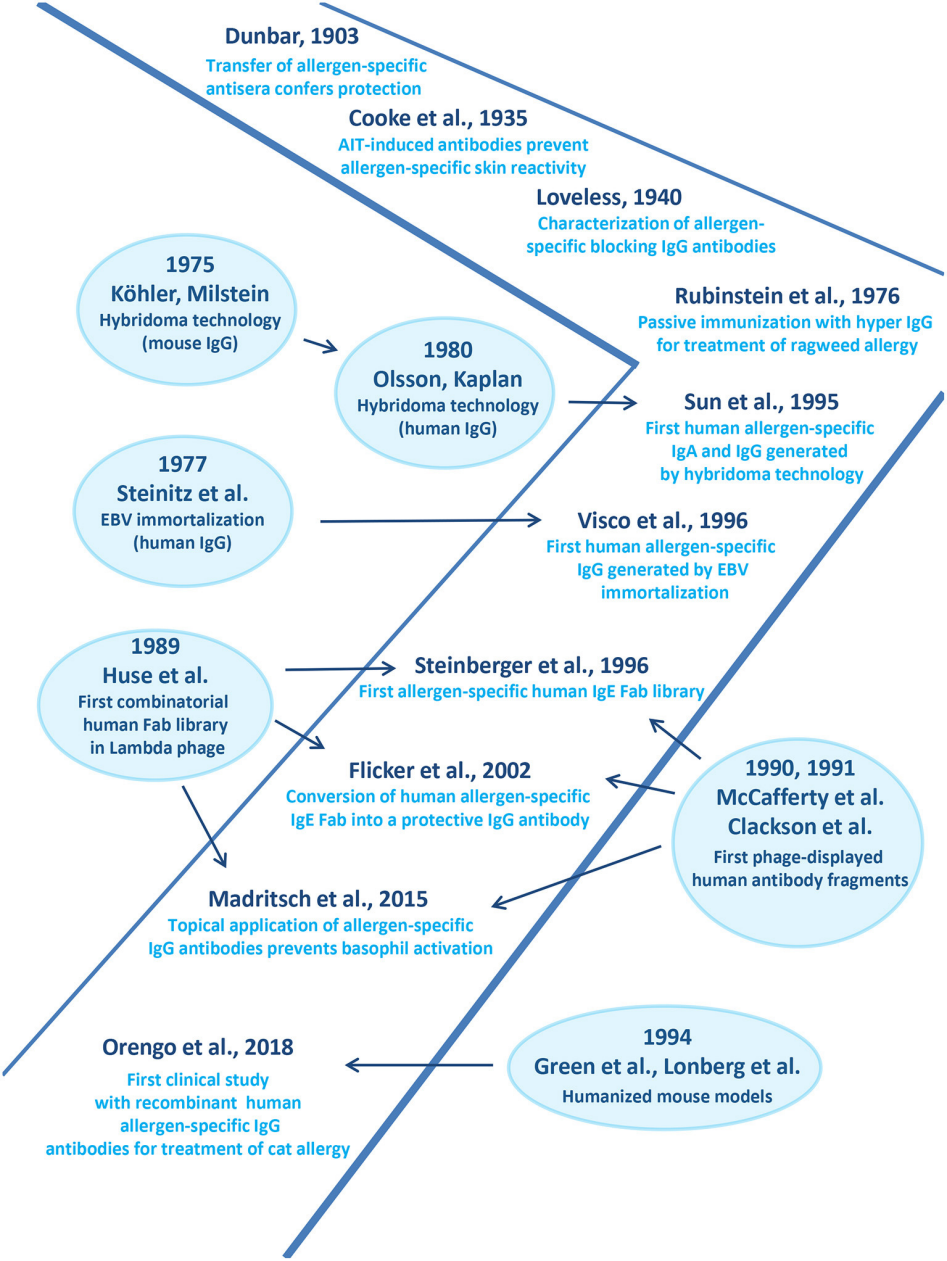
Important developments in the field of monoclonal antibody production accompanying advances made in treatment of allergy by human allergen-specific antibodies are shown.

Figure 2 provides a timeline of the studies highlighting the role of allergen-specific blocking IgG for treatment of allergy in the context of technological advances made toward the production of recombinant specific monoclonal human antibodies in general. The first evidence that immune-sera raised in animals against grass pollen allergen extract protect against allergic inflammation originates from a paper by Dunbar (194) (Figure 2).

Then R.A. Cooke and co-workers published their experiments demonstrating that immune-sera from AIT-treated patients suppressed allergen-specific skin reactivity in human subjects (Figure 2) (195). Loveless showed that blocking antibodies prevent allergen-IgE recognition (Figure 2) (196). She further demonstrated an association between the amount of protective antibodies and clinical improvement of AIT and identified IgG antibodies as major isotype involved in blocking (196, 197). The importance of allergen-specific IgG for treatment of allergy was corroborated by the demonstration that passive immunization of allergic patients with IgG derived from non-atopic volunteers who had been immunized with large doses of ragweed-extract protected against allergen-induced inflammation (Figure 2) (198).

### Monoclonal Antibodies in Allergy Treatment

A milestone toward the development of monoclonal antibodies was the invention of hybridoma technology by G. Köhler and C. Milstein which allowed production of mouse monoclonal antibodies on a large scale (Figure 2) (199). Their method was further utilized to generate human IgG antibodies (Figure 2) (200). In parallel, Steinitz and associates established human lymphoid lines immortalized by Epstein-Barr virus (EBV) transformation for the production of antibodies with defined antigenic specificity (Figure 2) (201). The introduction of these technologies enabled the generation of human monoclonal IgA and IgG antibodies specific for the major ragweed allergen, Amb a 1(202) and shortly thereafter, of human IgG antibodies specific for the major birch pollen allergen, Bet v 1 (Figure 2) (203) One of the Bet v 1-specific monoclonal IgG antibodies strongly inhibited IgE binding to Bet v 1 and Bet v 1-induced basophil degranulation and thus was considered a candidate for treatment of birch pollen allergy (203).

In order to be able to generate libraries of antibodies resembling the specificities of a complete organism the combinatorial library technology was developed. This technology was based on the isolation of cDNAs coding for the heavy and light chains from the antibody producing host, their random combination to obtain all possible pairs of heavy and light chain combinations and the isolation of specific antibody fragments (Fabs) or single chain fragments (ScFvs) (204–206). The combinatorial library technology actually allowed for the first time isolation of human allergen-specific IgE and provided access to their variable region sequences (Figure 2) (207) conversion of a grass pollen allergen-specific IgE Fab into a complete human IgG antibody it could be shown that this technology can be used to obtain human monoclonal IgG antibodies which block allergic inflammation *in vitro* highlighting their therapeutic potential antibodies (Figure 2) (208).

This human grass pollen allergen (Phl p 2)-specific blocking antibody was then further developed for topical application as described in Figure 1B (193). A bispecific conjugate consisting of the Phl p 2-specific IgG and a monoclonal ICAM-1 specific antibody was shown to anchor the conjugate on the surface of a respiratory epithelial cell layer and to prevent the transmigration of the allergen and subsequent allergen-induced inflammation underneath the epithelium (193).

In order to obtain large numbers of allergen-specific human IgG antibodies for treatment, the company Regeneron has used a technology which allows generation of panels of human IgG antibodies by immunization of mice. This technology is based on transgenic mice containing complete human antibody repertoires (Figure 2) (209, 210). Based on the first generation of such transgenic mice, further HumAbmouse approaches were established, e.g., VelocImmune mice for the efficient production of fully human antibodies (211, 212). Using this refined technology two fully human IgG4 antibodies specific for Fel d 1, the major cat allergen were generated recently and shown to be effective for the treatment of cat allergy in a clinical trial (Figure 2) (213). This proof of principle study showed that a single subcutaneous injection of a mixture of these two human monoclonal IgG4 antibodies significantly reduced allergic symptoms in cat allergic patients and the effect of treatment lasted for ~3 months (213). This study thus suggested that treatment by passive immunization with allergen-specific IgG which blocks allergic patients IgE binding to the culprit allergen can be an effective treatment for allergy but there are limitations of this approach.

### The Basis for Allergy Treatment by Passive Immunization With Monoclonal Allergen-Specific IgG Antibodies and Its Limitations

The mechanisms of action of the monoclonal antibodies used for treatment by passive immunization are similar to those in AIT (191, 213). AIT induces by active vaccination a polyclonal allergen-specific IgG response which competes with the patients' IgE for allergen binding by occupying the epitopes recognized by IgE. As a result of this competition, intruding allergens are captured by IgG and thus cannot trigger IgE-mediated mast cell or basophil activation, they fail to induce IgE-facilitated allergen presentation to T cells and do not boost systemic IgE production (214–217). This leads to a reduction of immediate allergic symptoms, T cell-mediated allergic inflammation and eosinophil recruitment as well as of allergen-specific IgE production (218). It is obvious that polyclonal allergen-specific IgG induced by AIT is more effective in blocking the binding of the polyclonal IgE to the allergen than single monoclonal allergen-specific IgG antibodies. For certain allergens such as the major birch pollen allergen, Bet v 1 it was possible to identify single monoclonal IgG antibodies which potently blocked the polyclonal Bet v 1-specific IgE in the majority of birch pollen allergic patients (203). For the major cat allergen Fel d 1 a profound blocking of cat allergic patients polyclonal IgE to Fel d 1 was achieved with a cocktail of two monoclonal antibodies (213). However, there are several highly potent allergens such as the major grass pollen allergen, Phl p 5 which consists of two flexible IgE-reactive domains (219). For Phl p 5 it was not possible to inhibit patient's polyclonal IgE binding even by a cocktail of several monoclonal IgG antibodies (220). Phl p 5 is only one of the four clinically relevant allergens recognized by grass pollen allergic patients, which comprise in addition Phl p 1, Phl p 2, and Phl p 6 and account for a high percentage of grass pollen-specific IgE (221, 222). Accordingly, it will be very difficult, if not impossible, to define a small-enough panel of grass pollen allergen-specific monoclonal IgG antibodies which are capable of blocking the majority of grass pollen allergen-specific IgE. The same is true for other important allergen sources such as house dust mites in which six important allergens (i.e., Der p 1, Der p 2, Der p 5, Der p 7, Der p 21, and Der p 23) have been identified (223). Regarding cat allergy it is clear that Fel d 1 is the most important allergen, but several other cat allergens have been identified (e.g., Fel d 2, Fel d 3, Fel d 4, Fel d 5, Fel d 6, Fel d 7, Fel d 8) (224). Their clinical relevance has not yet been defined but it is quite likely that blocking Fel d 1-specific IgE alone will be insufficient to treat all cat allergic patients.

Considering that many allergic patients are sensitized to several independent and antigenically unrelated allergen sources it will be difficult to create cocktails of therapeutic monoclonal antibodies which cover the necessary range. This problem exists for AIT which can be used mainly for treatment of patients who have a limited number of clinically-relevant driving allergens.

One possibility to obtain a large panel of monoclonal allergen-specific IgG antibodies resembling a polyclonal IgG cocktail for difficult allergens and complex allergen sources is to make use of humanized mouse models. This will be technically challenging. Alternatively, one can consider immunizing healthy subjects with defined allergen molecules to generate therapeutic immunoglobulin G preparations which are enriched for polyclonal blocking allergen-specific IgG antibodies. In this context it should be mentioned that it was recently demonstrated that non-allergic subjects could be safely immunized with recombinant allergen derivatives to induce polyclonal allergen-specific IgG which strongly blocked allergic patients IgE binding to the corresponding allergen (225). In fact, immunization of non-allergic subjects with hypoallergenic recombinant Bet v 1 was safe and did not induce allergic sensitizations in the vaccinated subjects and the induced IgG antibodies blocked polyclonal IgE binding to Bet v 1.

Another possibility to render treatment with allergen-specific IgG antibodies more feasible would be topical application of the antibodies with the goal to prevent them from passing through the epithelial barrier. Accordingly, allergens would be captured “outside” of the epithelial barrier and would not reach underlying mast cells and T cells (Figure 1B). Therefore, for capturing and keeping allergens “outside” one could eventually use monoclonal antibodies which do not compete with allergic patients' IgE binding to the allergen. Experimental evidence for such an approach is outlined below.

### Topical Application of Monoclonal Allergen-Specific IgG Antibodies for Treatment of Allergic Rhinitis

Topical application of drugs for the treatment of allergy represents a first line treatment for rhinitis, conjunctivitis, asthma and dermatitis. For each of the target organs sophisticated devices for drug delivery have been developed and are available. It is therefore tempting to speculate that topical administration of therapeutic allergen-specific blocking antibodies to the nose for treatment of allergic rhinitis could be an alternative to systemic passive immunization. However, there are a few hurdles which need to be overcome. First of all, topically applied antibodies will be quickly washed out by nasal secretions and the mere formation of allergen-antibody immune complexes will not completely prevent allergens from passing the epithelial barrier. Accordingly, it will be important to build up a shield of protective antibodies on the outer surface of the respiratory epithelium which stays there long enough so that only one or two applications per day are necessary to keep the antibody shield intact. To prevent washing out of topically applied antibodies we therefore considered immobilizing them to ICAM-1 which is a molecule that is highly expressed on the surface of airway and conjunctival epithelial cells in allergic patients and which has a low surface turn-over (226, 227).

In a proof of principle study we generated antibody conjugates bispecific for ICAM-1 and the major grass pollen allergen Phl p 2 by biotin-streptavidin coupling of a monoclonal anti-ICAM-1 antibody and a Phl p 2-specific human monoclonal IgG antibody) (193). We found that the conjugate remained immobilized on the surface of a layer of cultivated respiratory epithelial cells and prevented the allergen from transmigration through the cell layer. The allergen transmission was reduced substantially so that basophil activation with allergen-containing culture medium collected from the basolateral side of the epithelial layer was strongly reduced as compared to that from the apical side (193).

These proof of principle experiments thus demonstrated that immobilization of allergen-specific IgG on epithelial cell layers via ICAM-1 has the potential to prevent trans-epithelial allergen migration and to reduce allergic inflammation in the underlying tissue. These experiments were carried out with chemical conjugates of the two monoclonal antibodies but there are a variety of possibilities to generate bi-specific antibodies or alternative scaffolds of different formats in different expression systems in a quality suitable for clinical application, in sufficient quantities and at reasonable costs to make the topical treatment affordable (228). Moreover, we conducted further experiments in which we used a monoclonal ICAM-1-specific antibody which blocks the binding of major group RVs to ICAM-1 and a monoclonal allergen-specific IgG antibody (229) which did not block IgE binding but has high affinity for the allergen. We found that this conjugate strongly prevented RV infection and in addition could trap the allergen on the apical side of the epithelial cells and prevent allergic inflammation at the basolateral side (230). This result indicates that it may be possible to perform topical treatment with one high affinity monoclonal antibody per allergen without need for a cocktail of IgE blocking antibodies. Keeping the allergen outside the epithelium may be sufficient to prevent allergic reactions. Moreover, the use of ICAM-1 antibodies capable of blocking major group RV binding to respiratory epithelial cells may at the same time prevent RV infections.

We therefore propose topical treatment with ICAM-1 anchored allergen-specific IgG antibodies or scaffolds as alternative to passive immunization with allergen-specific IgG antibodies. Table 2 summarizes features of the two forms of treatment. Passive immunization will be only effective when IgG antibodies are used which compete with IgE antibodies for allergen binding because they cannot prevent the allergen from crossing the epithelial barrier. By contrast, topical administration of ICAM-1 anchored antibodies can be performed with non-IgE-blocking antibodies because the bispecific conjugates prevent allergen from trans-epithelial migration and thus keeps the allergen outside. Accordingly, one allergen-specific IgG per allergen may be sufficient for topical treatment whereas cocktails of IgE-blocking antibodies will be necessary to cope with complicated allergens and complex allergen sources. Passive immunization requires full length antibodies expressed in mammalian cells with long half-life whereas topical treatment can be performed with small molecules which can be obtained by relatively inexpensive expression in Escherichia coli. However, passive immunization confers long-term protection for months whereas topical treatment will need to be carried out at least once per day. Passive immunization can be used only for treatment of allergy whereas topical application of conjugates bispecific for allergens and ICAM-1 may protect against allergy and certain RV infections.

**Table 2:** Passive immunization with or topical application of monoclonal allergen-specific IgG antibody for treatment of IgE-mediated allergy.

**Passive immunization**	**Topical application**
Requires IgE blocking antibodies	May be performed with single non-IgE blocking antibodies
Requires full antibodies with long serum half-life	Can be done with antibody fragments or small scaffolds
Works only for certain less complex allergens and allergen sources	Can be used for complex allergens and allergen sources
One systemic administration sufficient for up to 3 months	Daily topical administration
Only for allergy treatment	Suitable also for treatment of RV infections with a blocking ICAM-1 antibody

The technologies for realizing both forms of antibody-based treatment are available and it will hence be possible to bring them into clinical trials. However, it remains to be seen whether any of the antibody-based forms of treatment is clinically more effective than currently available pharmacotherapy.

## Conclusions

The work described in this review is exciting- and some of it, such as LAIV to prevent influenza, has already provided improvements in human health. Interferons, beneficial bacteria, and bispecific antibodies are promising, but require further trials before being translated into clinical care. Despite the potential importance of the digestive tract in regulating immune responses, results remain controversial for orally-administered probiotics. While several murine studies have demonstrated that probiotics may have beneficial effects in CRS, there are no consistent results in human CRS trials. Responder groups may be hidden within the large diversity in endotypes and phenotypes of CRS. Fortunately the ready accessibility of the nose enables application of materials, in contrast to those which necessitate injection. A nasal vaccine for COVID- 19 would probably speed the delivery of relief from the pandemic. Intranasal therapy is likely to be a growth area.

## Author Contributions

GS conceived the idea of reviews on the nose as a route for therapy and commissioned the sections of this part 2 paper on intranasal immunotherapy. GS edited the paper, with help from SF, YP, and SL, and wrote the abstract and conclusion. PT wrote on intranasal vaccination. SF and RV contributed the work on bispecific nasal antibodies. YP, JC, OK, and SJ have written the Interferon part. SL, AE, and MD have written the Probiotics section. All authors contributed to the article and approved the submitted version.

## Conflict of Interest

AE has participated in advisory boards for ALK Abello, AstraZeneca, Aralez, Bausch Health, Circassia Ltd, GlaxoSmithKline, Johnson & Johnson, Merck, Mylan, Novartis, Pediapharm and Pfizer, has been a speaker for ALK, Aralez, AstraZeneca, Boerhinger-Ingelheim, CACME, Meda, Mylan, Merck, Novartis, Pediapharm, Pfizer, The ACADEMY, and Takeda. Her institution has received research grants from Bayer LLC, Circassia Ltd, Green Cross Pharmaceuticals, GlaxoSmithKline, Sun Pharma, Merck, Novartis, Pfizer, Regeneron and Sanofi. She has also served as an independent consultant to Allergy Therapeutics, Bayer LLC, Ora Inc. and Regeneron in the past. GS reports fees and consultancies from ALK, Mylan, Bayer, GSK outside the submitted work. The remaining authors declare that the research was conducted in the absence of any commercial or financial relationships that could be construed as a potential conflict of interest.

## Abbreviations

IFN: interferon
AIT: Allergen-specific Immunotherapy
EBV: Epstein-Barr virus
Fab: antigen binding fragment
HumAb mice: transgenic mice that produce fully human antibodies
ICAM-1: Intercellular Adhesion Molecule 1
RV: rhinovirus
ScFv: single chain Fragment variable
LDLR: Low density lipoprotein receptor
CDHR3: Cadherin-related family member 3
SARS-CoV-2: the virus causing COVID-19.
